# Differential arousal and neural engagement for angry and fearful faces: a combined pupillometric and fMRI study

**DOI:** 10.3389/fnhum.2025.1739802

**Published:** 2026-01-12

**Authors:** Kim C. Wende, Roman Kessler, Kristin M. Rusch, Jens Sommer, Andreas Jansen

**Affiliations:** 1 Department of Psychiatry and Psychotherapy, University of Marburg, Marburg, Germany; 2 Institute of Child and Adolescent Psychiatry, University of Kiel, Kiel, Germany; 3 Core-Facility Brainimaging, Faculty of Medicine, University of Marburg, Marburg, Germany

**Keywords:** emotion quality, face processing, fMRI, gestalt, occipital cortex, perceptual load, pupillometry, superior temporal cortex

## Abstract

**Introduction:**

Understanding how emotions are encoded at the neural level remains a central challenge in human neuroscience. Facial expressions are among the most powerful and frequently used stimuli to study emotion processing. Face perception itself is a complex function supported by a core network—including bilateral occipito-fusiform and superior temporal regions—and an extended network involving anterior structures such as the bilateral amygdalae. However, previous findings on how emotional content modulates these networks have been inconsistent.

**Methods:**

To disentangle perceptual and affective components of face emotion processing, we combined high-frequency pupillometry with functional magnetic resonance imaging (fMRI). Pupillary dilation serves as a sensitive index of two distinct processes: perceptual load, reflecting the informational complexity of a face, and arousal, indicating its immediate sensory impact. In our study, 25 participants (13 female) viewed faces expressing anger, fear, happiness, or neutrality as well as luminance-matched houses serving as control stimuli. A one-back task unrelated to emotion masked the true experimental purpose.

**Results:**

Relative to houses, faces elicited stronger pupillary dilations as well as enhanced blood-oxygen-level-dependent (BOLD) activity in bilateral occipital and fusiform cortices as well as in both amygdalae. Among facial expressions, angry faces evoked the largest pupillary dilations, while fearful faces elicited the strongest neural responses within a right-lateralized network centered on the superior temporal sulcus (rSTS). Across all faces>houses (conjunction minimum-statistic inference), pupil size correlated positively with BOLD activity in the right fusiform gyrus (rFFG), left inferior occipital gyrus (lIOG), bilateral calcarine cortex, and bilateral lingual gyrus.

**Discussion:**

These findings indicate that emotional faces impose a higher perceptual load than matched control stimuli, engaging a distributed network spanning early visual and attention-related areas. In conclusion, our results suggest that emotional quality is specified early in the perceptual process, with divergent pupillary and neural signatures separating arousal-driven threat responses (anger) from socially complex alarm cues (fear).

## 1 Introduction

The quality of an emotion constitutes a complex set of informational features. In the visual domain, the human face represents the most salient and universally used medium for expressing emotions. Across cultures, observers reliably categorize a limited set of basic emotions from facial expressions (Ekman, 1992). Yet it remains an open question whether the human brain performs a similar classification of facial emotions—and which neural regions integrate emotional quality into perceptual and experiential representations. FMRI studies have established that face processing engages a core network, encompassing the bilateral occipital and fusiform gyri as well as the superior temporal cortex, and an extended network, including anterior structures such as the bilateral amygdalae (Haxby et al., 2000, 2002; Fairhall and Ishai, 2007). Recent meta-analytical work supports the notion of the core and extended face processing networks even for dynamic stimuli (Zinchenko et al., 2018).

How exactly emotional expressions are represented within this network remains an open question in both computationally inspired and fundamental neuroscience. It is still debated whether emotional information is embedded within the holistic (Gestalt) representation of a face or constitutes a distinct perceptual cue. Lesion evidence particularly implicates the right fusiform gyrus in processing individual facial identity (Rossion, 2008), whereas emotion processing has been more broadly linked to amygdala function—extending beyond facial stimuli (Davis and Whalen, 2001).

In fMRI research, the focus has traditionally been on the type of information being processed—the “what”—rather than on the dynamics of processing—the “how” (e.g., Fusar-Poli et al., 2009a, 2009b). In contrast, evidence from a recent event-related potential study comparing bodily and facial expressions suggests that the automatic processing of emotional signals from the body influences face recognition, but not as strongly in the opposite direction (Puffet and Rigoulot, 2025). Phenomenologically, the faster processing of body compared to facial emotions may reflect the fact that bodily cues more directly indicate possible actions and are therefore, as sensory data, more immediately relevant to the perceiver (de Gelder, 2006). In short, whereas engineered, action-unit–based models infer emotional categories from discrete cues such as facial muscle configurations, the human brain implements a more complex, valence-guided, and feedback-sensitive coding scheme that continuously biases activity in early visual areas (Murphy et al., 2011; Deen et al., 2015).

Moreover, the processing of movement and continuity—the temporal integration of dynamic changes in facial and bodily signals—plays a crucial role in social perception. Within the core face network, the superior temporal cortex has been identified as the key site for integrating social and motion-related cues, thereby supporting both social-cognitive interpretation (Blakemore, 2008) and visuomotor processing (Grosbras et al., 2012).

Conceptually, the term *emotion quality* remains under-specified in face perception research. Most paradigms in the field still follow Ekman’s framework of basic emotions (Ekman, 1992; see Vytal and Hamann, 2010). This model distinguishes classes of negative (e.g., fear, anger) and positive (e.g., happiness) valence; yet, what constitutes *successful processing* of such emotions remains unclear. For instance, autism research frequently operationalizes social cognition through Ekman-based facial emotion recognition tasks (Nagy et al., 2021). Paradoxically, the sheer abundance of studies using Ekman faces has produced rather inconsistent behavioral and neural results. A central conceptual debate concerns whether a baseline emotion truly exists—that is, whether any facial expression can be regarded as genuinely *neutral* and emotionally unloaded (Uljarevic and Hamilton, 2013). At the neurophysiological level, emotional valence interacts with visual salience (or arousal) (Corbetta and Shulman, 2002). In subjective experience, both implicit salience and explicit valence jointly shape the perceived Gestalt of a face.

Pupil dilation offers a valuable window into the neural mechanisms underlying emotion perception. Two complementary concepts are particularly informative:

- Perceptual load, which reflects the cognitive demands of stimulus processing and has been linked to emotional valence (Kahneman and Beatty, 1966; Kahneman and Wright, 1971; Kahneman et al., 1969).
- Arousal-based neural responses, which precede non-arousal-related processes and correspond to sensory salience (Honma et al., 2012; Murphy et al., 2011; Tamietto et al., 2009).

In neuroimaging studies of emotional face perception, high-frequency pupillometry can help disentangle these processes by distinguishing rapid, arousal-driven responses from slower, cognitively mediated perceptual load effects. Traditional pupillometry shows that the time course of dilation encodes stimulus salience (Kret et al., 2013), whereas the slower component of the pupil response—emerging later and reflecting the processing demands of complex visual stimuli—can be captured with high temporal precision using high-frequency eye-tracking (Wierda et al., 2012). The simultaneous acquisition of high-frequency pupillometry and fMRI is methodologically critical for a holistic neurophysiological account of face perception, as it bridges a fundamental resolution gap. fMRI’s low temporal resolution, on the order of seconds, is ill-suited to capture the rapid, sub-second dynamics of the subcortical visual pathways-including the superior colliculus and the pulvinar-that are intimately engaged in the initial, arousal-related components of processing socially salient faces. Pupillary oscillations, controlled by these same autonomic brainstem circuits, provide a continuous, millisecond-scale readout of this rapid arousal response. By correlating this high-fidelity temporal trace of arousal witch the spatially precise hemodynamic response signal from fMRI, researchers can disambiguate the distinct, yet temporally intertwined, contributions of the fast, subcortical arousal network from the slower, higher-order cortical regions involved in detailed face analysis, thereby providing a more complete and directionally linked model from initial orienting to full cognitive appraisal.

We therefore combined fMRI (MRI data entered a connectivity analysis whose results are already published; see, Kessler et al., 2021) with high-frequency pupillometry to identify distinct neural mechanisms underlying face perception and the processing of emotional quality in response to Ekman (1992) expressions. We focused on anger and fear, as previous work suggests that these emotions differ in their underlying quality and functional significance (Davis et al., 2011). We hypothesized that angry faces, as direct threat signals, would evoke greater pupil dilation, reflecting heightened arousal, whereas fearful faces, which indicate environmental alarm, would preferentially engage the right superior temporal sulcus (rSTS)—a region implicated in the integration of social and motion cues. To ensure balanced emotional valence, happy and neutral expressions were included as comparison conditions (Uljarevic and Hamilton, 2013). Luminance-matched house images served as non-social control stimuli.

## 2 Methods

### 2.1 Subjects

Twenty-five healthy volunteers (13 female; age range 21–29 years, mean = 24.3, SD = 2.1), recruited from students and staff at the University of Marburg, participated in the study. All participants were right-handed (Oldfield, 1971), had normal or corrected-to-normal vision, and reported no history of neurological or psychiatric disorders. Written informed consent was obtained from all participants. Experimental procedures were conducted in accordance with the Declaration of Helsinki and approved by the local Ethics Committee (proposal #30/16).

### 2.2 Experimental design

**Figure 1:**
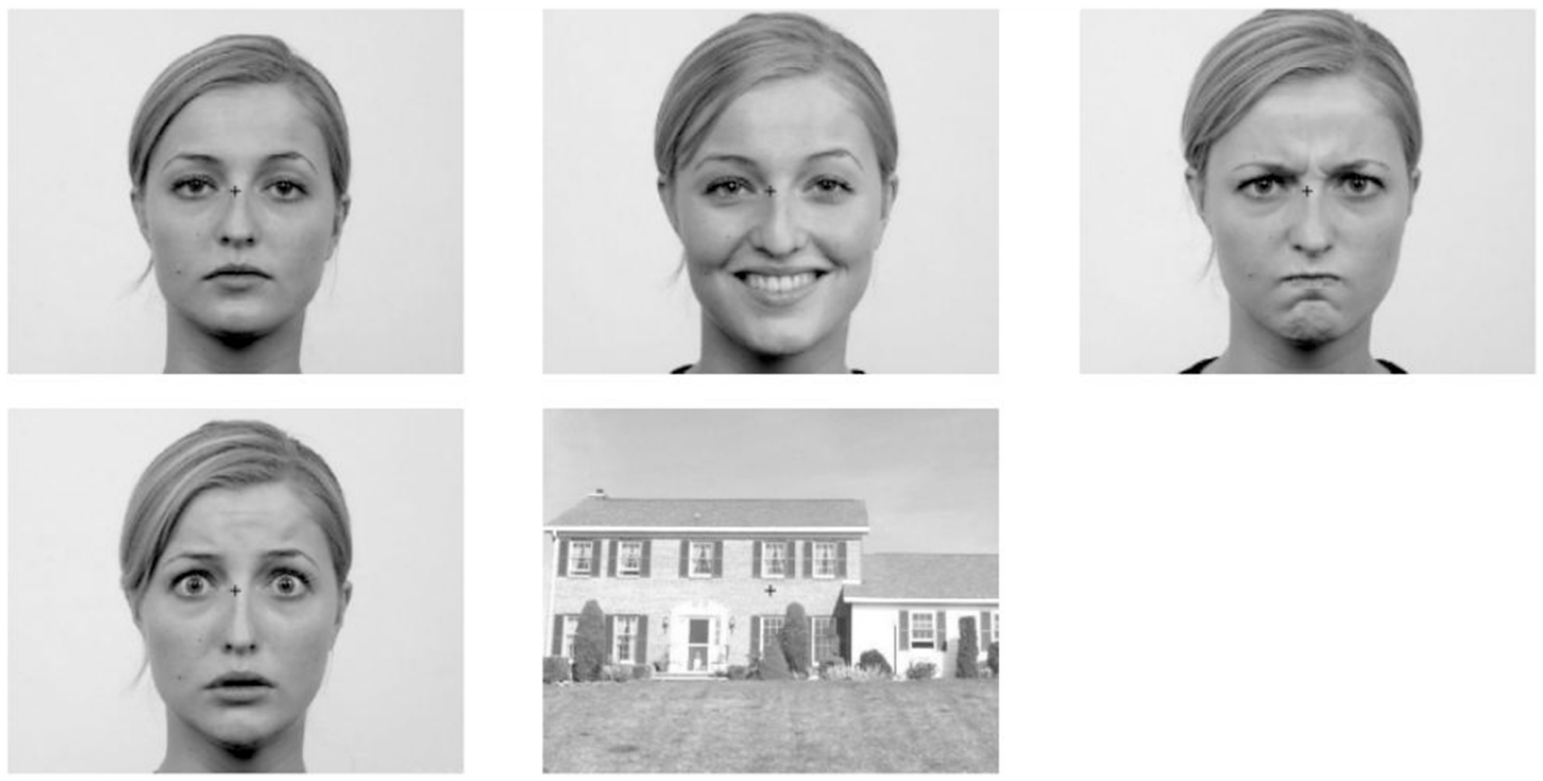
Example stimuli. Faces displaying neutral (top left), happy (top middle), angry (top right), and fearful (bottom left) expressions, alongside luminance-matched houses as a control condition (bottom middle). Faces reproduced with permission from Langner et al. (2010).

**Figure 2:**
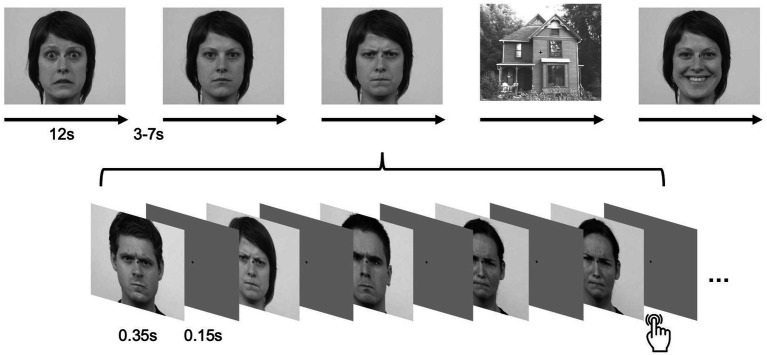
Procedure of the experiment. Each stimulus is presented for 350 ms, with an inter-stimulus interval of 150 ms. Twenty-four images form a block with a duration of 12 s. Between blocks, there is a break lasting between 4 and 7 s. Twenty blocks from each category (neutral, happy, angry, fearful, houses) are presented in a pseudo-randomized order. If the same stimulus is repeated, the participant should indicate the repetition by pressing a key. Faces reproduced with permission from Langner et al. (2010).

Five stimulus conditions were presented: faces displaying neutral (NF), happy (HF), angry (AF), or fearful (FF) expressions from the Radboud Faces Database (Langner et al., 2010), and houses (H) as a control condition. All images were converted to grayscale and cropped to 500 × 400 px using ImageMagick (version 6.8.9–9, Q16 x86_64; ^©^1999–2014 ImageMagick Studio LLC). Mean luminance was equated across stimuli using the SHINE toolbox for MATLAB (Willenbockel et al., 2010). Spatial-frequency matching was deliberately omitted in order to preserve the natural frequency content that is critical for rapid face and emotion processing via subcortical pathways (Vuilleumier et al., 2003). Because pupil responses are highly sensitive to even subtle changes in low- and mid-frequency structure, any artificial SF equalization would have compromised the ecological validity of the stimuli as well as the perceptual mechanisms underlying the pupillary signal. In addition, all stimuli were already luminance-matched and have been validated in previous work; further SF manipulation would likely have introduced distortions that run counter to the aim of presenting perceptually natural emotional stimuli. Example stimuli are shown in Figure 1. Experimental Procedure is shown in Figure 2.

Stimuli were presented in alternating block conditions (NF, HF, AF, FF, H) on a rear-projected 16:9 monitor, viewed via a mirror positioned approximately 15 cm above the participant’s eyes in the MRI scanner (Presentation v14.1, Neurobehavioral Systems). Participants were naive to the experimental purpose and performed a cover one-back task (button presses with both index fingers) to maintain attention.

Each block contained 24 stimuli, each displayed for 350 ms with 150 ms inter-stimulus intervals. The block order was identical for all participants. In total, 100 blocks (~12 s each) were presented, yielding an experimental duration of ~30 min. Each block was preceded by a fixation cross presented for a jittered interval of 4,000–7,000 ms.

### 2.3 Data acquisition

#### 2.3.1 Pupillometry data

Left-eye pupil diameter was recorded continuously at 1 kHz during each ~12 s block using an MRI-compatible EyeLink 1,000 infrared camera (SR Research). A 5-point calibration was performed prior to recording. Blinks were identified using the standard EyeLink detection routines.

#### 2.3.2 MRI data

MRI data was acquired on a 3 T Siemens scanner (TIM Trio, Siemens, Erlangen, Germany). High-resolution T1-weighted anatomical images were acquired for each participant using a magnetization-prepared rapid gradient-echo (3D MP-RAGE) sequence in sagittal orientation (TR = 1900 ms, TE = 2.54 ms, voxel size = 1 × 1 × 1 mm^3^, 176 slices, 1 mm thickness, flip angle 9°, matrix size = 384 × 384, FoV = 384 × 384 mm). Functional data were collected using a T2*-weighted EPI sequence sensitive to the BOLD contrast (TR = 1,550 ms; TE = 36 ms; flip angle = 70°) with 20 transverse slices (slice thickness = 2.7 mm; interslice gap = 0.4 mm; FoV = 200 mm; voxel size = 2.8 × 2.8 × 3.1 mm, including gap). This sequence was chosen based on pilot data to provide robust single-subject amygdala activation.

### 2.4 Data analysis

#### 2.4.1 Pupillometry data

Blinks and saccades were detected using EyeLink routines with standard thresholds (saccade acceleration ≥ 500°/s^2^; velocity ≥ 50°/s). Microsaccades were treated as saccades. Segments containing blinks within the first 1,500 ms of a block were excluded due to presumed reduced attention at block onset. For the included trials/blocks, blink periods were linearly interpolated (Frässle et al., 2016). To reduce sequence effects, pupil traces were normalized per block to the average pupil size during the first 200 ms following the first stimulus onset (Wierda et al., 2012). Preprocessing and temporal analyses were performed in MATLAB (R2014a).

Pupil traces from 0–5 s relative to the first stimulus onset were extracted for each condition. This window captures both fast (initial) and slow (later) responses, while remaining within the first half of the block, which is assumed to be less affected by blink-related artifacts. The remaining ~7,000 ms of each block were excluded due to increased noise from blinks.

Mean pupil dilation within the 0–5 s window was compared between conditions using Wilcoxon–Mann–Whitney tests with sequential Bonferroni correction (*α* = 0.05). In addition, an ANOVA (SPSS 21, IBM) was conducted to assess the effect of condition on mean pupil dilation.

Parametric modulation of fMRI by pupil size: Parametric regressors were derived from the initial 5 s of each block. Pupil data were normalized to baseline (0–200 ms) as percentage change, downsampled to match the MR micro-time resolution, and demeaned for SPM compatibility. Regressors were then convolved with the canonical hemodynamic response function and resampled at the TR (1.55 s). These parametric regressors were included per condition as effects of interest in a second first-level fMRI model.

#### 2.4.2 MRI data

MRI data were preprocessed using SPM12 (r6685; Wellcome Centre for Human Neuroimaging; MATLAB). The first three functional volumes were discarded to allow for T1 signal stabilization. Field maps were computed from phase and magnitude images, converted to voxel displacement maps, and used to unwarp EPI images. A combined realign-and-unwarp procedure corrected for static and motion-related susceptibility distortions, while within-subject motion was further corrected using 6-parameter rigid-body transformations. Functional images were then normalized to Montreal Neurological Institute (MNI) space and smoothed with a 6 mm full-width-at-half-maximum (FWHM) Gaussian kernel.

A general linear (Generalized Linear Model, GLM) block design was specified for the five conditions (NF, HF, AF, FF, H) using the canonical HRF without temporal or dispersion derivatives. Onset vectors were generated for each participant from Presentation logs. The six motion parameters were included as nuisance regressors. A high-pass filter at 1/256 Hz was applied (extended from the standard 1/128 Hz). For each participant, condition-specific effects produced five t-contrasts corresponding to NF, HF, AF, FF, and H.

For the pupil-covariation GLM, conditions were modeled as regressors of no interest, while t-contrasts targeted the parametric modulators derived from the initial-phase pupil data. To isolate face-selective correlations, each face condition was contrasted against houses at the first level, resulting in four t-contrasts per participant (pupilmod_NF: NF > H, etc.).

At the group level, a flexible factorial model was used to combine single-subject contrasts. Unless otherwise noted, second-level contrasts were evaluated using t-statistics with voxel-wise family-wise error (FWE) correction (*p* < 0.05) and a cluster-extent threshold of k ≥ 10 voxels, to reduce false positives in small ROIs such as the amygdalae. For the anatomical labelling of resulting cluster peak voxel location, the SPM-implemented anatomy toolbox (atlas) was used.

##### 2.4.2.1 Contrasts of interest

Commonalities and differences in emotional face processing: a group conjunction of all face > house contrasts (NF > H ∩ HF > H ∩ AF > H ∩ FF > H) was used to assess shared face-related BOLD responses. Differences among the two negative emotions were examined using pairwise contrasts (i.e., FF > AF, AF > FF). To assess the common negative valence of fear and anger, we computed for each the conjunction contrasts to the two non-negative conditions (FF > HF) ∩ (FF > NF), (AF > HF) ∩ (AF > NF).

Parametric modulation by pupil dilation: a main-effect t-contrast tested the average modulation of face-related BOLD activity by pupil dilation over 0–5 s (pupilmod_NF, pupilmod_HF, pupilmod_AF, pupilmod_FF), with family-wise error correction applied.

## 3 Results

### 3.1 fMRI data

#### 3.1.1 Activation for faces across all emotions

**Figure 3:**
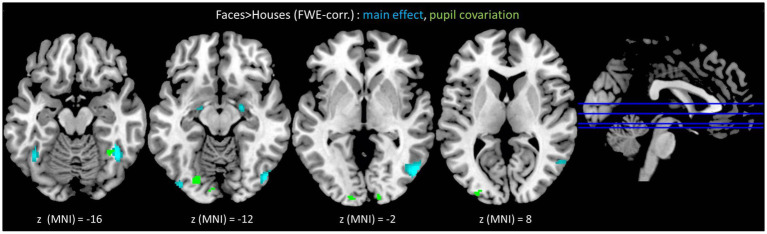
Turquoise: face-related brain activation, irrespective of emotional content [i.e., conjunction contrast (neutral faces > houses) ∩ (happy faces > houses) ∩ (angry faces > houses) ∩ (fearful faces > houses)], was observed in the bilateral IOG and FFG —the core face perception network—as well as in the bilateral AMY. Green: associations between pupil dilation and BOLD activity were found in multiple regions of the occipito-temporal cortex, including the core system of face perception as well as more posterior located regions in the early visual cortex. Statistical threshold: *p* < 0.05, FWE-corrected, with a cluster-extent threshold of 10 voxels. IOG = inferior occipital gyrus, FFG = fusiform gyrus, AMY = amygdala.

The conjunction of all face > house contrasts (NF > H ∩ HF > H ∩ AF > H ∩ FF > H) revealed increased BOLD activity in the bilateral inferior occipital (IOG) and fusiform (FFG) gyri, corresponding to the core face perception network, as well as in the bilateral amygdalae (AMY) (Figure 3, turquoise; Table 1).

**Table 1:** fMRI results for the conjunction contrast (neutral faces > houses) ∩ (happy faces > houses) ∩ (angry faces > houses) ∩ (fearful faces > houses).

Cluster	MNI-coordinates (x, y, z)			Cluster size	Peak T-value	Cluster *p*-value
R FFG	42	−52	20	111	10.79	<0.001
R IOG	48 54	−72 −52	−6 4	360	10.20 5.80	<0.001
L FFG	−42	−54	−18	80	8.85	<0.001
L IOG	−40	−84	−12	28	6.88	<0.001
R AMY	22	−6	−12	23	6.55	<0.001
L AMY	−18	−8	−14	12	5.48	0.002
	16	−36	20	31	5.53	<0.001

Statistical threshold: *p* < 0.05, FWE-corrected, with a cluster-extent threshold of 10 voxels. FFG = fusiform gyrus, IOG = inferior occipital gyrus, AMY = amygdala, R = right, L = left.

#### 3.1.2 Differential activations of negative emotions

Significant differences were observed only for fearful faces (Table 2). The conjunction FF > HF ∩ FF > NF revealed clusters in the right superior temporal sulcus and gyrus (STS/STG), right IOG and left IOG. The contrast FF > AF showed increased responses in the right IOG, right STS, and right amygdala (AMY).

**Table 2:** fMRI results for the differences between negative emotions.

Cluster	MNI-coordinates (x, y, z)			Cluster size	Peak T-value	Cluster *p*-value
Contrast: (fearful faces > happy faces) ∩ (fearful faces > neutral faces)						
R STS	48	−42	6	168	7.20	<0.001
R IOG	36	−84	−8	31	5.65	0.015
R STG	52	−60	−2	17	5.38	0.035
L IOG	−20	−86	−14	15	5.24	0.045
Contrast: fearful faces > angry faces						
R IOG	32	−88	−8	81	6.06	0.001
R STS	42	−36	10	32	5.90	0.012
R AMY	26	0	−14	20	5.53	0.025

Significant increases of BOLD-response were observed only for fearful faces. Statistical threshold: *p* < 0.05, FWE-corrected, with a cluster-extent threshold of 10 voxels. STS = superior temporal sulcus, IOG = inferior occipital gyrus, STG = superior temporal gyrus, AMY = amygdala, R = right, L = left.

### 3.2 Pupillometry data

**Figure 4:**
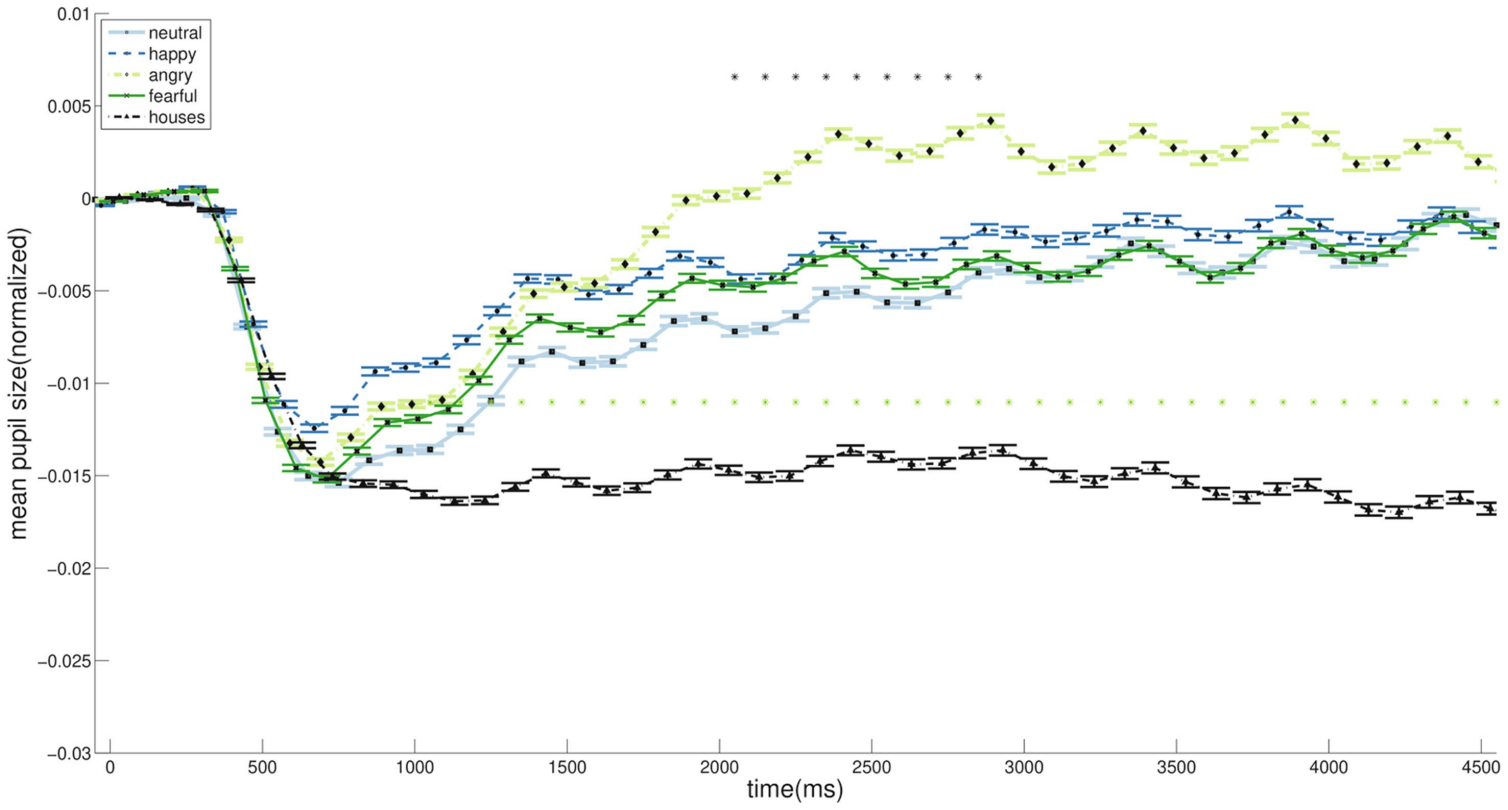
Averaged pupil dilations over time (normalized to the first 200 ms of each block; 0–5,000 ms after first stimulus onset). Following stimulus onset, the pupil initially constricted, peaking at approximately 600 ms, and subsequently redilated. Redilation was significantly larger for faces than for houses (light stars). Among face conditions, angry faces elicited a significantly greater increase over time compared with the other expressions (dark stars).

Initial pupil constriction peaked at approximately 600 ms after the first stimulus onset, followed by redilation, which reached its maximum from around 2,500 ms onward. After the initial constriction, all face conditions exhibited larger pupil sizes over time compared with houses (Figure 4, light stars). A repeated-measures ANOVA revealed a significant main effect of condition on pupil dilation, *F* = 14.8, *p* < 0.001, partial η^2^ = 0.059. A sensitivity power analysis conducted in G*Power 3.1 for a within-subjects ANOVA with five measurements, using a sample size of 25, *α* = 0.05, and 80% power, determined that this design could detect effects of size *f* ≥ 0.22, which is below our observed effect size. Notably, ranksum test revealed that angry faces elicited a significantly greater increase over time than all other face conditions (Figure 4, dark stars). Bonferroni post-hoc tests revealed that pupil dilation was significantly greater in AF compared to NF (mean difference = 0.0081, *p* < 0.001), HF (mean difference = 0.0055, *p* = 0.011), FF (mean difference = 0.0065, *p* = 0.001) and H (mean difference = 0.0168, *p* < 0.001), while H elicited significantly smaller dilation than all other conditions (mean differences H-NF = −0.0087, H-HF = −0.0113, H-FF = −0.0103, all *p* < 0.001).

### 3.3 Combination of pupillometry and fMRI data

Parametric analyses revealed a significant main effect of parametric modulation by pupil dilation on face-emotion-specific BOLD activity in the left inferior and middle occipital gyri (IOG, MOG), the right fusiform (FFG), as well as in more posterior regions such as the left occipital pole (OCP), the right calcarine gyrus (CAL), and the bilateral lingual gyrus (LG) (Figure 3, green; Table 3).

**Table 3:** Main effect of pupil dilation on BOLD responses for neutral, happy, angry, and fearful faces.

Cluster	MNI-coordinates (x, y, z)			Cluster size	Peak T-value	Cluster *p*-value
R FFG	34	−50	−16	27	6.58	<0.001
L IOG	−22	−80	−12	45	6.44	<0.001
R CAL	14	−96	−2	19	6.26	<0.001
L MOG	−30 −25	−92 −85	8 12	41	5.95 5.88	<0.001
L LG	−10	−88	12	18	5.72	<0.001
R LG	26	−78	−6	16	5.69	<0.001
L OCP	−14	−98	−2	19	5.49	<0.001

Statistical threshold: *p* < 0.05, FWE-corrected, with a cluster-extent threshold of 10 voxels. FFG = fusiform gyrus; IOG = inferior occipital gyrus, MOG = middle occipital gyrus; CAL = calcarine cortex; OCP = occipital pole; LG = lingual gyrus; R = right; L = left.

## 4 Discussion

The perception of emotion from faces integrates both sensory input and cognitive appraisal. In this study, we examined how the perceptual load of emotional faces, indexed by pupillometry, relates to their neural processing, with a particular focus on dissociating pathways for the negative emotions of anger and fear.

### 4.1 The perceptual load of faces

Consistent with the established core face-perception network (Haxby et al., 2000, 2002), faces evoked greater BOLD responses in bilateral occipital and fusiform cortices and larger pupillary dilations than luminance-matched houses. This is in line with evidence for face-selective attentional modulation under high perceptual load (Neumann et al., 2011).

Critically, the parametric modulation of the BOLD signal by pupil dilation provides direct evidence for this link, showing that face-evoked pupil time courses correlated with activity in a distributed occipital network including the right fusiform gyrus (rFFG), left inferior (lIOG) and middle occipital gyrus (lMOG), the left occipital pole (lOCP), and crucially, early visual areas such as the bilateral calcarine (CAL) and lingual gyri (LG).

The pupil-linked modulation in the calcarine cortex is particularly informative. As a site of primary visual processing (Klein et al., 2000) that is modulated by attention and behavioral relevance (Han et al., 2005), its correlation with pupil size—a known index of arousal and processing demand (Kahneman and Beatty, 1966; Bradley et al., 2008)—strongly suggests that faces impose a higher perceptual load than inanimate objects.

This load-related activity extends into the lingual gyrus, a region associated with internally directed attention and known for early face-selective responses (Benedek et al., 2016; Paré et al., 2023), supporting its role in forming the abstract representation of the face category (Watson et al., 2016). These findings indicate that the core face network, particularly the right fusiform gyrus, is not only engaged for face processing per se, but that its activity level is tuned to occipital-lingual representations of overall perceptual load, as reflected in pupil diameter.

### 4.2 Anger dilates: a threat-triggered arousal response

Our key finding reveals a clear dissociation between anger and fear: while anger specifically enhanced pupil dilation, fear preferentially engaged distinct neural regions. This suggests that anger processing is characterized by a broad, arousal-dominated response.

Anger likely drives this heightened perceptual effort due to its direct threatening nature, whereas fear signals an indirect, environmental threat. This aligns with findings that angry faces are better remembered, suggesting they draw attention to the threatening agent itself, whereas fear directs attention outward to the environment (Davis et al., 2011). Our pupillometric data indicate that this anger-specific response is rapid, with a stronger pupil response emerging between 1800–2,900 ms—a timeframe compatible with late affective appraisal in event-related potential (ERP) studies (Klein et al., 2015). This rapid arousal response likely biases early visual processing (Vinck et al., 2015), priming the system for immediate action.

From a Gestalt perspective, visual systems prioritize cues with immediate behavioral relevance. The direct threat of potential violence conveyed by anger is thus prioritized, triggering a global arousal state reflected in the pupil. This dovetails with work showing angry faces modulate frontal empathy networks (Enzi et al., 2016). The fact that the amygdala was more engaged by fear than anger further underscores this dissociation; the amygdala’s role in vigilance for ambiguous threats (Davis and Whalen, 2001) makes it more critical for processing the alarm signal of fear than the clear, direct threat of anger.

### 4.3 Fear engages: a neural signature for social alarm

In contrast to the broader arousal response elicited by anger, fearful faces recruited a circumscribed and right-lateralized network encompassing the superior temporal sulcus (STS), inferior occipital gyrus (IOG), and the amygdala. Fearful expressions selectively increased activation in the right STS and IOG relative to happy and neutral faces, and—critically—engaged the right STS and amygdala more strongly than anger.

This pattern suggests that fear processing extends beyond basic threat detection, engaging circuits specialized for decoding socially informative cues. The STS is a well-established hub for integrating dynamic facial features, biological motion, and gaze direction (Deen et al., 2015; Grosbras and Paus, 2006), all of which are essential for identifying both the source and direction of potential danger. Recent evidence further indicates that rapid visual pathways supporting fear detection may already encode high-level social information rather than merely low-level threat signals (e.g., Lanzilotto et al., 2025). This interpretation aligns with contemporary work emphasizing that the amygdala contributes not only to vigilance but also to the evaluation of ambiguous or context-dependent social stimuli (Davis and Whalen, 2001).

In essence, while anger tends to trigger a direct “body alarm” reflected in peripheral autonomic responses such as pupil dilation, fear preferentially engages a “social-cognitive alarm” that mobilizes the STS and amygdala to search for the source of threat in the environment.

## 5 Conclusion and synthesis

In summary, our multimodal approach dissociates the neural and psychophysiological pathways for processing angry and fearful faces. We demonstrate that anger is predominantly associated with a threat-triggered arousal response, indexed by pupil dilation, which reflects a global state of preparedness. In contrast, fear is characterized by the specific engagement of a right-lateralized network—including the STS and amygdala—specialized in processing social cues and environmental alarm. This “anger dilates, fear engages” dichotomy provides a parsimonious framework for understanding how the brain efficiently processes distinct negative emotional qualities to guide adaptive behavior. We particularly consider the role of a fast, subcortical pathway (involving the superior colliculus, pulvinar, and amygdala) in the rapid processing of fear. This “low road” provides a mechanistic foundation for the amygdala’s rapid, automatic response to fearful faces, which then initiates a vigilant state and guides subsequent cortical analysis (de Gelder et al., 2011). In this framework, the direct threat of anger may be less dependent on this rapid subcortical alert. Instead, anger processing might engage cortical pathways more directly from the outset, supporting the detailed appraisal of hostile intent and coordinating the broad, sustained cortical arousal reflected in the pupil dilation.

### 5.1 Limitations

The interpretability of our findings is subject to several design constraints. Conceptually, whether the higher perceptual load is due to the no-emotion-neutrality of faces is an interpretation of the pupillary modulation findings that needs to be verified. A replication of the combined high-frequency pupillometric and fMRI study using only neutral face stimuli, would serve this purpose. Methodically, our strategic choice to optimize for robust subcortical and ventral temporal coverage resulted in a limited field of view (20 slices), potentially omitting activity in higher-order regions such as the prefrontal cortex. Additionally, the fixed block design, while powerful, precludes the disentanglement of transient neural responses from sustained emotional adaptation and may be susceptible to order effects. Leaving spatial frequencies natural preserves the ecological validity but might have confounded results. Future studies manipulating spatial frequencies, in particular in relation to the amygdala response, would help address this question. Finally, the use of a one-back cover task, though effective for controlling attention, may have inadvertently modulated emotional processing through its added cognitive load.

### 5.2 Outlook

The distinct “arousal-for-threat” versus “engagement-for-alarm” model we propose provides a clear, testable framework for future research. Crucially, these findings underscore the necessity for replication in independent cohorts, particularly to confirm the robustness of the right STS in fear processing. Our study also highlights the advantage of a multimodal approach. Relying solely on fMRI might have led to the simplistic conclusion that fear is “more processed” than anger in temporal regions, whereas pupillometry alone would have suggested anger is the more potent stimulus. It was only by combining these measures that we could dissociate the broad, arousal-based impact of anger from the specific, socially-informative neural engagement elicited by fear. Future studies should leverage this multimodal strategy to investigate whether this dichotomy generalizes to other stimuli, such as dynamic faces or full-body expressions, and to explore its potential alterations in clinical populations with deficits in threat or social cue processing.

## Data Availability

The raw data supporting the conclusions of this article will be made available by the authors, without undue reservation.
