# Correction: Differential arousal and neural engagement for angry and fearful faces: a combined pupillometric and fMRI study

**DOI:** 10.3389/fnhum.2026.1804299

**Published:** 2026-02-18

**Authors:** Kim C. Wende, Roman Kessler, Kristin M. Rusch, Jens Sommer, Andreas Jansen

**Affiliations:** 1 Department of Psychiatry and Psychotherapy, University of Marburg, Marburg, Germany; 2 Institute of Child and Adolescent Psychiatry, University of Kiel, Kiel, Germany; 3 Core-Facility Brainimaging, Faculty of Medicine, University of Marburg, Marburg, Germany

**Keywords:** emotion quality, face processing, fMRI, gestalt, occipital cortex, perceptual load, pupillometry, superior temporal cortex

Author Andreas Jansen was omitted as corresponding author. The correct corresponding authors are Kim C. Wende, kim.wende@staff.uni-marburg.de; Andreas Jansen, andreas.jansen@staff.uni-marburg.de.

The original version of this article has been updated.

